# Advancing Evidence-Based Nursing: The Updated German Expert Standard on Continence Promotion

**DOI:** 10.3390/healthcare13212771

**Published:** 2025-10-31

**Authors:** Julien Pöhner, Julia Kaiser, Moritz Krebs, Andreas Büscher, Daniela Hayder-Beichel

**Affiliations:** 1 Department of Social Science, Bremen University of Applied Sciences, Am Brill 2–4, 28195 Bremen, Germany; 2 Department of Healthcare, Niederrhein University of Applied Sciences, Reinarzstr. 49, 47805 Krefeld, Germany; 3 Department of Economics and Social Sciences, Osnabrueck University of Applied Sciences, Albrechtstr. 30, 49076 Osnabrueck, Germany

**Keywords:** expert standard, urinary incontinence, fecal incontinence, evidence-based care

## Abstract

Background: Incontinence is a widespread and socially taboo phenomenon worldwide. Incontinence, with its various manifestations, is one of the most common illnesses in outpatient medical care and represents a serious health problem for those affected of all ages. As part of the second update of the German expert standard published in 2024 on continence promotion, a systematic literature review was conducted to identify, appraise, and synthesize current evidence on nursing interventions to promote urinary and fecal continence. The expert standard does not provide a gradation of recommendations, but rather that the criteria depicted in the standard have the highest possible recommendation character in the sense of the best available knowledge. The aim of this article is to present the examination of available evidence within the context of the second update of the expert standard. Methodology: A systematic literature review was conducted between September and December 2022 with additional guideline research in December 2023 in Medline (via PubMed), CINAHL (via EBSCO), and the Cochrane Library, using predefined inclusion and exclusion criteria. Additional guideline databases and organizational websites were searched manually. The review process and reporting were guided by PRISMA 2020 reporting standards. Eligible studies included qualitative, quantitative, and guideline publications in English or German published since 2012. Study selection, data extraction, and critical appraisal were conducted independently by two reviewers. Results: Of 2850 initial records, 60 studies met the inclusion criteria and were included in the review. The majority were systematic reviews and evidence-based guidelines. The central literature-based results of the expert standard are presented based on the steps of the nursing process. The findings were thematically synthesized along the steps of the nursing process and informed key nursing interventions for continence promotion and compensation, including assessment, patient education, pelvic floor training, and selection of continence aids. Discussion: There are a variety of evidence-based interventions that can be used to deal with urinary and/or fecal incontinence and the tasks that professional nurses take on in promoting continence are complex. Patients and their relatives want information and advice on treatment options, reliable contacts and individual support offers to make informed decisions.

## 1. Introduction

Incontinence is a globally prevalent and socially taboo phenomenon [1,2]. Incontinence, with its various forms, is one of the most common illnesses in outpatient medical care and represents a serious health problem for those affected of all ages [3].

The prevalence of urinary and fecal incontinence increases with age, with women being affected more commonly due to anatomical factors and experiences of childbirth [4]. From an international perspective, it is difficult to define clear prevalences. But in Germany, depending on the definition, an estimated 8% of adults are affected by urinary incontinence and 1–15% by fecal incontinence. Approximately 0.5–1% suffer from regular fecal incontinence [5,6].

In acute inpatient care, 24% of patients suffer from urinary incontinence and 13% from fecal incontinence. In long-term care, approximately 70% of residents are affected by urinary incontinence and 40% deal with fecal incontinence [7,8]. Given its high and increasing prevalence across healthcare settings, incontinence represents a major clinical and societal challenge that requires coordinated, person-centered responses from the nursing profession [9,10].

The consequences of incontinence are diverse and can include a significant reduction in health-related quality of life, as well as physical, psychological, and social effects [11]. As the diagnosis, treatment and compensation for incontinence incur substantial costs, the economic consequences are significant for both those affected individuals and the health care system [3,12,13]. However, due to shame and fear of stigmatization, but also the assumption that incontinence is part of the aging process, only a quarter of men and women with urinary and/or fecal incontinence seek professional help, e.g., in nurses [6,12]. Therapeutic options are available, although compared to surgical procedures in clinical practice conservative therapies often have a lower risk of harm [2]. Implementing these therapies is often entrusted to caregivers, who, however, may experience uncertainties in care due to various factors (such as limited specific knowledge about the affected groups, increasing complexity of care, staffing shortages, or a lack of evidence-based practices in nursing) [6,14]. Nevertheless, nurses in primary health care are a strategic pillar in early detection and management. Nurse specialists undertake traditional and effective tasks in complex care situations, for specific patient groups (e.g., those with lower urinary tract symptoms or overactive bladder), or in specific continence-promoting measures [15]. Guidelines provide bundled evidence-based knowledge regarding urinary and fecal incontinence and thus support a structured, patient-oriented approach. However, guidelines often have a special focus in created by physicians for physicians. Nursing professionals are rarely addressed as users and original nursing guidelines are rarely found.

To address this gap in nursing-specific guidance, the German Network for Quality Development in Nursing (DNQP) develops since 1992 expert standards tailored to the needs and responsibilities of professional nurses.

The aim of this article is to present the examination of the available evidence within the context of the second update of the expert standard, as a nationally recognized, evidence-based guideline developed specifically for nursing practice with the aim to define a high, science-based level of care across all healthcare setting published in 2025 “Promoting urinary continence in nursing care” that in its updated version will be called “Promoting continence in nursing care”.

In contrast to the expert standard itself, this article presents the results of the evidence base and methodological principles underlying the updated standard. It contributes to the scientific discourse by disclosing the process and criteria for evaluating, synthesizing, and translating evidence into practice recommendations. It thereby creates transparency and provides a transferable model for similar international standard development efforts. Furthermore, this article explicitly addresses an international academic audience by providing insights into the methodological approach and scientific evidence that underpins the German expert standard. This article examines the evidence base and methodological principles underpinning the updated expert standard, with the aim of promoting transparency and supporting the international transfer of evidence-based nursing practice.

### Germany: The Importance of Expert Standards

The DNQP is an association of specialist colleagues that has been developing expert standards on relevant nursing topics across all sectors since 1992 with the aim of promoting nursing quality (https://www.dnqp.de/ (accessed on 23 October 2025)). Topics included pressure sores and falls prevention, pain management or oral health. Expert standards are defined as “evidence-based, monodisciplinary instruments that highlight the specific contribution of nursing to the healthcare of patients or residents and their families, addressing central quality risks and providing a basis for continuous improvement of nursing quality in healthcare and nursing facilities” [16] p. 7. As part of a professional, multi-stage coordination process, the aim is to integrate a large number of perspectives from clinical practice and nursing science into the performance level.

“The structure of the expert standards shows a matrix that horizontally identifies structure, process, and outcome criteria, and vertically follows the steps of the nursing process method from information gathering to evaluation. “Thus, expert standards encompass the entire process of risk assessment, action planning, information, education and counseling, as well as coordination, implementation, and evaluation of interventions related to a nursing-relevant topic, fitting into the logic of the nursing process method” [16] p. 13. A significant difference from guidelines is that expert standards do not involve a gradation of recommendations; instead, the criteria depicted in the standard, in line with the best available knowledge, hold the highest possible recommendation character.

The methodological approach is based on the international rules for standard and guideline development. To this end, a team of nursing scientists is dedicated to literature research and analysis. Based on the results of this literature search, a group of eight to twelve experts from nursing practice and nursing science formulate recommendations for key nursing interventions. These are presented as structure, process and result criteria and presented to the wider nursing community and agreed upon. This is done during the development of an expert standard through a consensus conference and during updates through an internet-based consultation phase. Several hundred people take part in this discourse and contribute to a critical assessment of the expert standard recommendations. The expert standard will then be implemented in around 25 healthcare facilities to test the practical suitability and acceptance of the instrument. An update takes place every 5–7 years. In the process of updating an expert standard, newly identified evidence is systematically compared to the previous version to assess whether revisions or expansions are necessary. This comparison is guided by predefined clinical questions and quality appraisal of the literature. New findings are analysed to determine if they confirm, complement, or contradict previous recommendations. The updated version of the expert standard is therefore based not only on a fresh review of evidence but also on a critical methodological assessment of the existing standard. This ensures continuity while integrating advancements in research.

The first expert standard on the topic of “Promoting Urinary Continence in Nursing” was published in 2007 and revised for the first time in 2014 with the current state of evidence [17]. As part of this second update, the topic of urinary incontinence was supplemented with the thematic focus of fecal incontinence, which was motivated by a growing body of evidence on its prevalence, impact, and care relevance, particularly in geriatric and long-term care settings. Furthermore, stakeholders in previous implementation phases had repeatedly requested a broader conceptualization of continence to reflect the needs of practice more accurately. In addition, the updated literature review identified new evidence on several key aspects of continence care, including the use of digital assistive technologies, updated continence profiles to better capture both urinary and fecal incontinence, and psychometric tools for the standardized assessment of symptom burden and quality of life. These findings contributed to an expansion and refinement of the expert standard’s recommendations.

## 2. Materials and Methods

This systematic review followed a systematic mixed-methods review approach. This was considered as it allowed not only for mapping the breadth of existing evidence but also for classifying the methodological quality and practical relevance of included studies [18]. Such an approach enables a more structured presentation of findings and supports the formulation of practice-oriented recommendations. It includes the systematic identification, appraisal, and thematic synthesis of quantitative, qualitative, and guideline-based literature. The systematic review was not registered in PROSPERO due to its integration into the structured update process defined by the German Network for Quality Development in Nursing (DNQP), which includes both evidence appraisal and consensus development components. Nonetheless, the methodological principles of systematic reviews guided all stages of the process, and reporting follows the PRISMA 2020 Statement [19]. It includes the systematic identification, appraisal, and thematic synthesis of quantitative, qualitative, and guideline-based literature.

### 2.1. Search Strategy

At the beginning of the update process, a systematic literature search was conducted from September 2022–December 2022 with an update search for guidelines in December 2023 with no results for the pre-defined questions (Supplementary Materials). The review questions were developed in an iterative, consensus-based process in collaboration with the expert working group, based on the thematic structure of the nursing process and the specific aims of the expert standard update. Due to the broad and practice-oriented nature of the questions, which include qualitative experiences, intervention effects, and assessment strategies, the questions were derived directly from practical priorities and evidence needs identified in the standard development process. Searches were carried out in the databases Medline via PubMed, CINAHL via EBSCO (including EMBASE), the Cochrane Library, LIVIVO, and Google Scholar (for an overview search). Furthermore, in addition to electronic databases, we manually searched the websites of specialist organizations and repositories for relevant guidelines. These included the websites of the National Institute for Health and Care Excellence (NICE), the Scottish Intercollegiate Guidelines Network (SIGN), the Registered Nurses’ Association of Ontario (RNAO), the German Guideline Registry (AWMF), the German Society of Nursing Science (DGP), and the Cochrane Nursing Care Field. We used a block-building approach [20] with respective synonyms for searching titles and abstracts. After searching the single terms for each block, all terms of a single block were combined using the Boolean operator OR. Results from this step were then combined for the 2 blocks using the Boolean operator AND. For example, the search string in PubMed included: (“urinary incontinence” OR “fecal incontinence”) AND (“nursing” OR “continence promotion”) AND (“assessment” OR “intervention” OR “training”), limited to studies published since 2012 in English or German (an overview can be found here: https://www.dnqp.de/fileadmin/HSOS/Homepages/DNQP/Dateien/Expertenstandards/Kontinenzfoerderung/Kontinenz_Akt2024_Literaturstudie-Anhang.pdf (accessed on 22 October 2025)). All hits were recorded for each step and question.

### 2.2. Study Selection

All designs of guidelines, quantitative and qualitative original research, meta-analyses, systematic reviews, and other types of reviews that address urinary or fecal incontinence were included. Study protocols, expert statements or conference posters were excluded. Using defined inclusion and exclusion criteria (Table 1).

Title, abstract, and full-text screening were conducted independently by two authors (J.P. and J.K.). Screening started after a random sample of 163 titles and abstracts was screened with blinding to authors and journal titles, achieving a Cohen’s Kappa of 0.87, indicating a strong agreement [21]. Citations identified in the final search step were organized in an EndNote library. For the title and abstract screening, the web-based tool, Rayyan was used [22]. The included sources underwent a full-text analysis and were subject to critical analysis using assessment tools. In the first step, the search and evidence synthesis focused on identified guidelines. These were critical analysed using the DELBI–German guidelines assessment tool. The instrument consists of 8 domains (e.g., participation of interest groups, methodological rigor, general applicability), corresponds to the essential quality requirements of the AGREE instrument and was supplemented by detailed questions to present the methodology, content and implementation strategy.

Furthermore, in the next step, systematic reviews and selected individual studies were used. Systematic reviews and individual studies with a quantitative study design were assessed using the “Scottish Intercollegiate Guidelines Network (SIGN)” checklist evaluated [23]. Their aim is to identify aspects that have a significant impact on the risk of bias in the reported results and the conclusions drawn.

The evaluation of qualitative individual studies was conducted using “Critical Appraisal Checklist for Qualitative Research” from the Joanna Briggs Institute. This consists of three sections and ten individual questions, which are intended to carry out an initial screening of the research work, but also to focus more closely on the methodological rigor and the results generated. This allows the most accurate overview of the quality of the individual research papers to be achieved. One point is awarded for each question, so that a total of ten points can be achieved [24]. The results of the assessments are attached to the expert standard.

The restriction to studies published from 2012 onward was chosen to align with the date of the previous expert standard update and to ensure consideration of the most recent evidence base. The exclusion of participants under 18 years reflects the scope of the expert standard, which is focused on adult nursing care and interventions in adult populations.

### 2.3. Study Analysis

The research, full-text evaluation and data extraction were conducted by two reviewers (J.P. and J.K.) working independently. Following each step, a joint exchange took place to discuss the approach and results, aiming for the most accurate representation of hits and findings. The extracted data were synthesized thematically along the domains of the nursing process (assessment, intervention, evaluation). Coding and categorization were conducted iteratively by the two reviewers and validated in expert group discussions to ensure consistency and applicability to practice.

In preparation for the discussion with the expert working group, the evidence was presented in a coherent manner. The structure follows the logical layout of the expert standard. For the discussion with the experts, as well as for the later publication, the particular challenge was to combine the scientific study and guidelines with an understandable conceptual analysis of the topics relevant to action. This is of particular importance in order to combine and present the problems of nursing practice with the results of the analyzed studies in a way that guides action.

## 3. Results

### 3.1. Research and Study Selection

**Figure 1:**
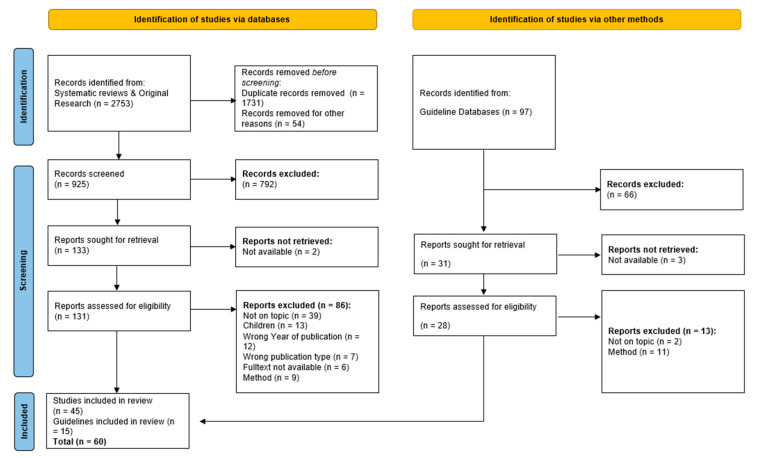
PRISMA 2020 flowchart of screening process and study selection.

Searches performed in databases identified a total of 2850 matches. The PRISMA flowchart in Figure 1 illustrates the study screening and selection process, indicating the numbers of records included and excluded. Initially, we removed 1828 duplicate records and 66 records for other reasons, resulting in 956 studies for title and abstract screening. Subsequently, 876 publications were excluded. After removing 20 studies due to methodological assessments, leaving 60 studies for inclusion in this review. While 60 studies met the inclusion criteria and were included in the full review process, not all of them are cited directly in this article. Some studies were used to support internal decision-making within the expert group and contributed to the overall synthesis but were not individually referenced due to thematic overlap or limited unique contribution.

### 3.2. Central Literature-Based Results

**Figure 2:**
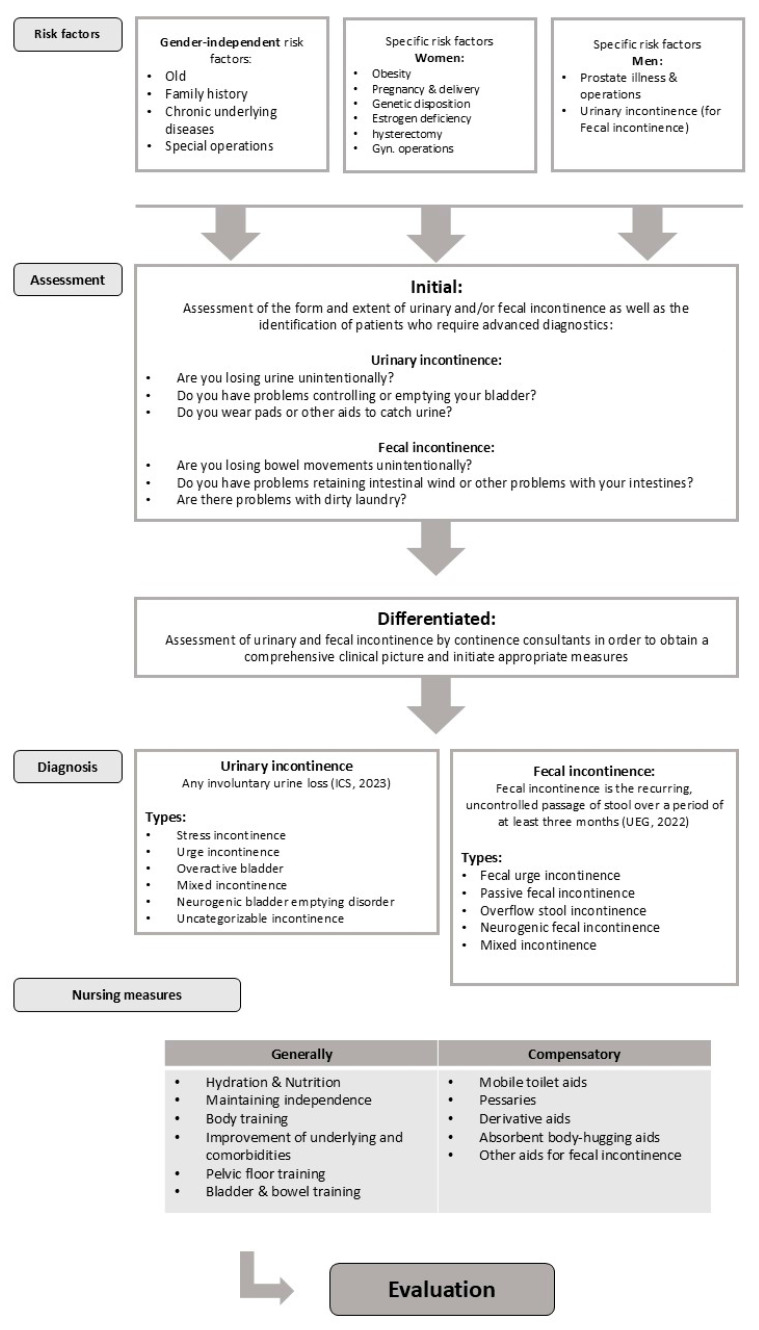
Process-based presentation of the literature-based results in the expert standard.

The central literature-based results of the expert standard are presented in alignment with the steps of the nursing process (Figure 2).

### 3.3. Assessment

The assessment is an important component in dealing with urinary and/or fecal incontinence. Therefore, symptoms and risk factors (Table 2) related to incontinence should initially be identified through screening [14]. A differentiated assessment is then made. In principle, all nurses should be able to carry out an initial and subsequently differentiated assessment. In-depth assessments, especially in complex care situations, are usually carried out through continence nurse specialists.

### 3.4. Initial Assessment of Urinary and Fecal Incontinence

Nurses should identify urinary and fecal incontinence promptly before seeking nursing care. Screening can be carried out using targeted questions (Table 3). The conversations should be conducted sensitively, and cultural preferences should be taken into account [5].

For an initial screening regarding the presence of incontinence, targeted questions are recommended [14,16].

If incontinence was detected in the initial assessment, a differentiated assessment of urinary and fecal incontinence is carried out by nursing staff or people with advanced continence expertise in nursing. This aims to obtain a comprehensive clinical picture and (derive and) initiate appropriate measures [5,12].

### 3.5. Differentiated Nursing Assessment of Symptoms and Effects of Urinary and Fecal Incontinence

Both urinary and fecal incontinence are multifactorial events caused by reversible and irreversible factors that should be considered systematically and, if possible, interdisciplinary. This becomes particularly clear when dealing with geriatric patients, as further diagnostics are often not desired, useful or feasible [14].

### 3.6. Anamnesis

The anamnesis is always the first and most important step in the differentiated assessment of urinary and fecal incontinence [1,2,3,5,6,26,27,28], which means that incontinence can be categorized and care planned in up to 80% of cases [3,12]. Significant aspects of the anamnesis for nursing professionals include fluid and dietary history, a survey of current medications, previous surgical procedures, previous and concomitant illnesses, functional limitations, as well as past treatments and management of existing incontinence. It is crucial to also capture the subjective burden of stress and the impact on the daily lives of the affected individuals.

### 3.7. Physical Examination, Urinalysis and Residual Urine Measurement

The physical examination, which should primarily be carried out by medical staff, is part of the initial assessment and serves to diagnose and classify treatment options for incontinence. It should always be carried out in a position that enables a reliable diagnosis and is associated with the best possible comfort for the patient. For example, through palpation of the abdomen, the detection of residual urine in the bladder as well as the detection of abdominal resistance or pain and an analysis of the urine, indications of the type of incontinence or risk factors for incontinence can be determined [1,2,3,12,13,14,27,28].

### 3.8. Micturition Protocol and Template Test

Micturition or bowel protocols provide, among other things, information on the frequency of urine and stool excretion using self- and external assessment [1,2,3,12,13,14,27,28]. Furthermore, a template test can be used to quantify the amount of urine loss, helping determine the severity of the incontinence and identifying a suitable aid [1,3].

### 3.9. Continence Profiles

Measures to promote continence are selected based on the goals, abilities and support needs of those affected. During the development of the expert standard “Promotion of urinary continence in nursing care, the expert working group at the time developed continence profiles [29]. They give nurses the opportunity to determine the degree of dependence on personal and/or material assistance in incontinent people and to evaluate the effect after nursing measures have been carried out. As part of the second update, the continence profile was expanded to include the topic of fecal continence (Table 4).

### 3.10. Recording the Symptoms and the Subjective Experience of Stress

The symptoms of urinary and fecal incontinence have an impact on the quality of life of those affected. To quantify the experience of stress, various questionnaires or scales should be used in a standardized manner before, during and after the therapies [1,13]. The questionnaires are differentiated according to their goals. There are symptom questionnaires, disease-specific ones and a combination of both, and specific as well as general questionnaires that are selected in a targeted and case-oriented manner [14]. In addition, international professional societies such as the ICS recommend the use of questionnaires at the assessment level. Validated questionnaires, including specific symptom values and voiding diaries, aid in screening and classifying the type of urinary incontinence [1,2,28].

There are more than 80 different questionnaires internationally [30], although only a few have a German translation. Additionally, there is a lack of psychometric testing on applicability, validity, and reliability for some of these instruments. Two instruments are recommended in German-speaking clinical practice: International Consultation on Incontinence Questionnaire on Urinary Incontinence–short form (ICIQ-UI short form) and, for a more in-depth analysis of health-related quality of life in urinary incontinence, the Kings Health Questionnaire (KHQ) [30,31].

There are different assessment systems in the area of fecal incontinence. Some of these systems have been validated, but none have found widespread use. The Cleveland Clinic Incontinence Score (CCS) was able to be identified and recommended in German translation for the standardized assessment of the frequency of fecal incontinence and the associated impairment of those affected [32].

### 3.11. “Yellow and Red Flags”

As part of the anamnesis, constant attention should be paid to so-called “red flags” and “yellow flags” (Table 5), which are considered alarm signals. These can provide indications of conditions where incontinence is only a symptom and not directly related to the diagnosis of incontinence [12,26].

In relation to fecal incontinence, the basic examination is described as a medical task [6]. The examiners must have the necessary skills to understand the subtypes of and be able to identify risk factors for fecal incontinence.

### 3.12. General Nursing Measures to Promote Continence

Nursing support for patients affected by incontinence should enable them to participate in social, leisure and cultural activities as well as education and work. General principles of patient-centered care must be taken into account [6].

In clinical practice, conservative therapies are recommended as a priority because they have the lowest risk of harm [2,28]. However, they should not be viewed in isolation but often represent part of a multi-component program [12]. It is essential that individuals’ needs are taken into account, and they are comprehensively informed about their own situation and intervention options for promoting continence [6]. Through targeted education and counseling, such as emphasizing that acceptable symptom control may only be achieved through the exploration of various therapeutic options, those affected are able to actively engage in the treatment process [13]. The expert standard provides in-depth descriptions of nursing interventions for primary care and continence nurse specialists, which are outlined below.

### 3.13. Hydration, Nutrition and Weight Loss

In the area of fluid intake, the recommendations from guidelines are rather cautious. Adjusting beverage choices or reducing fluid intake by 25% may help improve symptoms of overactive bladder but not urinary incontinence [2,3]. Restricting caffeine consumption may improve symptoms of urinary incontinence but has no effect on urinary frequency or urgency [34].

The nutritional recommendations relate specifically to fecal incontinence, as certain foods and diets can influence bowel activity. Therefore, a diet should be recommended that promotes ideal stool consistency and predictable bowel movements [5,6]. Keeping a food diary is recommended as effective [6].

The recommendations for weight loss refer to significantly overweight, predominantly younger women with urinary incontinence, aiming to reduce the frequency and improve global incontinence symptoms [2,14]. The reduced weight should be maintained in the long term [2,3].

### 3.14. Bowel Management

Recurrent constipation can have a negative impact on urinary and fecal incontinence. Despite little evidence, constipation prophylaxis is recommended by experts because there is a connection to the development of urinary incontinence [1,3,14].

### 3.15. Maintaining Independence/Environmental Adaptation/Toilet Adaptation/General Physical Training

In a home setting optimizing the living situation can have positive effects and minimize risks, such as floor coverings, without tripping hazards or improving lighting conditions [14]. Moderate physical activity is suggested to reduce the occurrence of urinary incontinence [1,34]. People with existing urinary incontinence should be encouraged to engage in low-intensity physical activity, provided it is tolerated by those affected.

### 3.16. Improvement in Underlying or Comorbidities

To improve urinary and fecal incontinence, it can be helpful to adequately treat the underlying and accompanying diseases (e.g., heart disease, diabetes mellitus, COPD, depression or neurological diseases) [12,28,34].

### 3.17. Smoking Cessation

The evidence on this topic is so far limited. There is weak evidence that smoking cessation can improve symptoms of overactive bladder, which is why smoking cessation strategies should be offered [1,2].

### 3.18. Pelvic Floor Training

Pelvic floor training can be carried out in a variety of ways. It is very difficult to compare the results [12]. The goal of pelvic floor training is to strengthen the periurethral and perivaginal muscles in women with stress incontinence, as strengthening the muscles can reduce incontinence symptoms [14]. Positive effects can be seen during pregnancy and in the postnatal phase. Exercise in early pregnancy is associated with a reduced risk of urinary incontinence in late pregnancy and the postnatal period [2].

For men, no clear recommendations can be made about the effectiveness of pelvic floor training. After radical prostatectomy, the EAU (2020) strongly recommends pelvic floor training. A cure could not be achieved through this intervention [1].

### 3.19. Bladder and Bowel Training

Bladder training is described as an effective intervention for improving urinary incontinence symptoms, with studies mainly examining female subjects [1,2]. Bladder training includes various behavioral interventions. In addition to going to the toilet at fixed or individual times, measures to manage the urge to urinate should also be included [12].

In the area of fecal incontinence, experts recommend bowel emptying training as equivalent to bladder training, but without evidence [6].

### 3.20. Nursing Measures to Compensate for Incontinence

Measures to compensate for incontinence are important nursing measures. The selection of suitable aids is based on practicality, benefits and possible risks for those affected [27]. It is particularly important to those affected that they are treated with sensitivity, especially regarding feelings of shame. Due to sometimes contradictory advice, there is often a lack of trust in the knowledge and skills of professional caregivers, and a lack of empathy is also criticized [6].

### 3.21. Pessaries for Women

Pessaries increase the stability of the urethra and enhance the resistance, resulting in improved continence rates and an increase in the quality of life. Pessaries can be used in addition to pelvic floor training, especially in women with an overactive bladder [1,2,3,12,34].

### 3.22. Mobile Toilet Aids

There is hardly any study on mobile toilet aids, which is why the use of these aids is based primarily on expert recommendations. Toilet aids should always be adapted to the individual needs of the user [14]. The use of raised toilet seats, grab bars, a commode chair or walking aids can enable patients with mobility limitations to achieve a targeted elimination situation [6,14].

### 3.23. Derivative Aids

The placement of a catheter is carried out strictly based on indications due to a voiding disorder, not for incontinence. A rigid residual urine volume alone is not a sufficient reason; rather, the combination of various factors must be considered (e.g., the presence of serious skin defects, the individual level of suffering or the need for export controls) [14]. Drainage systems are used permanently or intermittently, transurethrally or suprapubically and are specifically selected [27].

Intermittent (self-)catheterization (IK/ISK) promotes the influence of those affected on their urological health, their autonomy and the preservation of kidney function. Maintaining the number of daily catheterizations and infection prevention measures are crucial. In contrast, indwelling catheters are not recommended as a general treatment strategy due to the unfavorable benefit–risk ratio, as permanent drainage can be associated with an increased risk of urinary tract infections, urethral complications and impairments in everyday life [2,13,27,28].

When it comes to using derivative aids, detailed information and advice for those affected and their relatives is essential. Topics such as application in everyday life, advantages and disadvantages of the various options, possible complications and effects on everyday life, against the background of individual needs [14].

### 3.24. Absorbent Hygiene Products

There is a wealth of absorbent hygiene products, and according to current studies, no specific product recommendations can be made for urinary and fecal incontinence. One-size-fits-all approach should be rejected. The selection of products should be based on the following aspects: Adaptation of the material to the severity of the incontinence (as small as possible, as large as necessary!), preference for anatomically shaped products, products with optimal fluid retention (no pure cellulose products), material with odor absorption and as little noise as possible, and easy handling and secure fastening to prevent slipping [14].

### 3.25. Special Measures Fecal Incontinence

There are few special measures for those affected with fecal incontinence, e.g., anal plugs and fecal collectors. These aids are only limited and can be used for certain patient groups. Fecal collectors can be helpful in the intensive care setting for acute and severe diarrhea but are often poorly tolerated [6]. Anal plugs can also only be considered if accepted by those affected and as a second-line treatment for fecal incontinence [5]. Additionally, for individuals with persistently hard stools where other interventions do not improve the situation, transanal irrigation may be beneficial for patients with soft stool consistencies, the use of so-called stool bulking agents is recommended as part of a first-line treatment [5,6].

### 3.26. Advice

According to the recommendation of the Optimal Continence Services [15], nurses provide advice and engage in shared decision-making regarding general measures to promote autonomy and self-care, interventions for continence promotion, and compensation strategies. Especially for specific measures such as: B. bladder or pelvic floor training or instructions for self-catheterization, specialized nurses with expertise and a wide range of experience are involved (if possible in the facility).

### 3.27. Evaluation of Nursing Measures

There are a variety of nursing options available for promoting continence and compensating for urinary and fecal incontinence. The evaluation of nursing support should be carried out depending on the measures chosen and the target agreement. For example, the effect of pelvic floor training can only become apparent after a few weeks, whereas toilet training can be successful in a shorter period of time. The instruments of differentiated assessment must be used for the evaluation. With the information received and in exchange with the interdisciplinary team and those affected or relatives, decisions are made about the continuation or changes of the previous measures [17,25,35].

## 4. Discussion

This review demonstrates that conservative, nursing-led interventions remain central to effective continence promotion.

There are a variety of evidence-based interventions that can be used to deal with urinary and/or fecal incontinence and the tasks that professional nurses take on in promoting continence are complex. Patients and their relatives desire information and counseling on treatment options, reliable contacts and individualized support for informed decision-making [17]. The Expert Standard on this subject provides practical recommendations for the German healthcare practice to meet these needs. Furthermore, by presenting the methodological and conceptual synthesis behind the expert standard in an international context, this work makes the underlying evidence base more visible and transferable. It highlights how evidence-based nursing knowledge can be translated into practice guidelines and standards, providing an example for other countries or contexts where such structured nursing standards do not yet exist. While in Germany, the discussion about the importance of counseling within continence promotion has only taken place in recent years, there have been experiences internationally for decades. Therefore, it is not surprising that caregivers, for example, in the UK, the United States, Australia and Canada can rely on special qualification offers while in and Germany is still developing structured educational pathways. As a result, initial international study results illustrate the positive effect of specialized nursing advice [15]. In international comparison, there are significantly more scientific papers than in German-speaking countries.

In general, the majority of existing works in the fields of urinary and fecal incontinence are medically oriented; nurses are rarely described as users in guidelines and original nursing guidelines are hardly found. Nevertheless, in clinical practice, conservative therapies are preferred over surgical procedures if possible because they have a low risk of harm [2,28]. Despite the breadth of literature included, the limited number of high-quality, nursing-specific guidelines and qualitative studies represents a notable gap in the current evidence base. This lack of representation constrains the development of differentiated context-sensitive recommendations that adequately reflect the realities of nursing practice and the lived experiences of individuals affected by incontinence. Future research should aim to address this gap by promoting nursing-led guideline development, as well as conducting qualitative studies that explore the experiences, needs, and preferences of both patients and caregivers. Such research is essential to strengthen the evidence base for person-centered care and to support the professionalization of nursing within continence promotion. The implementation falls under the responsibility of nursing staff, and a multidisciplinary approach is recommended. The expert standard “Promoting Continence in Nursing” describes a professionally coordinated level of performance on the topic for professional nursing in all areas of care. Even if the expert standard does not follow the classic model of guidelines, it represents the same scientific point of view because it derives methodologically well-founded recommendations for nursing practice. In order to implement this level of quality, the involvement of professionals with the requisite skills and experience is necessary. These professionals should be able to provide advice, education, and support to those affected [6].

Expert standards can not only increase the quality of nursing care, but the economic perspective for health facilities and health care must also be considered. The introduction of an expert standard in the practice facilities is associated with a lot of time and costs, which, however, pay off economically. A German cost–benefit analysis reports costs of up to almost €60,000 for the introduction of the expert standard to promote urinary continence. However, these costs are weighed against the resulting benefits, such as improvements in the health status or continence profiles of those affected. The expert standard proves to be economically viable through savings and the proper use of incontinence materials. Through the introduction of the standard, the costs of incontinence materials were reduced significantly [36].

In addition to the economic considerations of implementing an expert standard, the effects on the quality of care are important. There are international efforts towards outcome measures to develop and test benchmark good care. There are publications on structure, process and result indicators [2,12,28,37,38] and a detailed set of 14 keys performance indicators, which were developed as the results of an expert panel [39]. The sources were used to formulate evidence-based quality indicators for Germany and to ensure regular quality measurement. A publication is also being planned after the implementation and evaluation of the results have been completed.

In addition to the current quality indicators, the expert standard also takes up new technical developments. The current publications discuss the use and status of digital assistance systems. The research on these systems is limited, and international guidelines have yet to extensively incorporate them. However, initial results show that systems for recording and assessing incontinence events or higher-level systems for promoting self-management with various interventions work well and are well-received. They have a positive impact on the quality of life, sleep quality, and fall occurrences of those affected, and they can contribute to a reduction in the workload of caregivers. Nonetheless, the production costs are high, and economic aspects must be critically examined [40,41,42,43,44]. Therefore, further research is necessary, including studies on the acceptance of these systems in nursing, their integration into existing nursing process-es, or the impact of technology on the nursing relationship.

In summary, it can be stated that the expert standard for continence promotion has achieved a high level of scientific and international standards in its development. It considers current developments in nursing and can have a structuring, participative, and economic impact on professional nursing, leading to measurable quality improvement.

## 5. Limitations

Naturally this work has limitations. One methodological limitation is the absence of a registered review protocol in PROSPERO. Although registration enhances methodological transparency and allows for independent verification of planned methods, the current review followed a predefined internal protocol as part of the structured expert standard update process. Therefore, while the review was systematic in its approach, external validation through registration was not possible due to procedural constraints. Furthermore, the literature search was limited to studies published in German and English, which may have excluded relevant findings published in other languages. Additionally, although the systematic literature search was conducted in late 2022, we have reviewed more recent publications to verify whether new guidelines or systematic reviews would alter the main conclusions. So far, no substantial updates have emerged, but a targeted update of the evidence base is planned to ensure continued relevance. Although qualitative studies were included and appraised using established tools, their overall representation in the evidence base remained limited. As a result, important experiential insights from those affected by incontinence may not have been fully captured. Additionally, the German-speaking research landscape remains predominantly medically oriented, with nursing professionals rarely addressed as primary users in existing guidelines. This lack of nursing-specific guidelines may have restricted the depth of nursing-specific recommendations. In the area of digital assistive technologies, the current evidence base remains limited, and recommendations are primarily derived from initial studies or expert opinions.

## 6. Conclusions

Despite the availability of evidence-based interventions, many affected individuals do not receive adequate care, which can be attributed to knowledge gaps, structural challenges, and an insufficient implementation of best-practice strategies. The updated expert standard establishes a high scientific benchmark for nursing practice and contributes to international discussions on evidence-based continence care. It provides a clear, scientifically grounded framework for nursing professionals to ensure a structured and patient-centered approach to continence care. It encompasses both preventive measures and specific nursing interventions aimed at promoting continence.

A particular emphasis is placed on the early identification of risk factors, interdisciplinary collaboration, and the role of specialized nursing professionals, who play a key role in counseling and providing care to affected individuals. The implementation and consistent application of the updated expert standards can make a significant contribution to improving the quality of care. It not only supports the professionalization of nursing but also helps maintain the independence and quality of life of individuals with incontinence while preventing unnecessary institutionalization or medical complications. To further strengthen the evidence base for nursing-led continence care, future research should prioritize the development of nursing-specific guidelines, should explore long-term outcomes of standard implementation and integration of digital assistive systems in routine care and qualitative studies that explore the lived experiences and care needs of affected individuals.

## Figures and Tables

**Table 1:** Inclusion and exclusion criteria for identifying scientific papers on incontinence.

Domain	Inclusion Criteria	Exclusion Criteria
**Urinary incontinence**		
Language	German English	Other languages
Study population	Over 18 years	Under 18 years
Years of publication	From 2012 ^1^	Before 2012 ^1^
**Fecal incontinence**		
Language	German English	Other languages
Study population	Over 18 years	Under 18 years

^1^ since the first update of the expert standard (2014) on the topic of urinary incontinence is already available and there is a systematic literature search in the year.

**Table 2:** Example representation of the symptoms of urinary and fecal incontinence [25].

Urinary Incontinence	Fecal Incontinence
Increased urinary frequency Nocturia Polyuria Involuntary loss of urine during physical charges Increased sensation of bladder filling Sudden urge to urinate Reduced sensation of bladder filling Lack of bladder sensation Delay in initiating emptying Compulsive urge to urinate Effort to defecate Weak urine stream Urine droplets at the end of voiding Dysuria (painful emptying of the bladder)	Decreased/increased rectal sensation Tenesmus (painful urge to defecate) Constipation Feeling of incomplete bowel emptying Straining during bowel movements Soiling after bowel movements Rectal bleeding/mucus

**Table 3:** Initial assessment questions to identify urinary and/or fecal incontinence [25].

Initial Questions About Urinary Incontinence	Initial Questions About Fecal Incontinence
Are you losing urine unintentionally? Controlling or emptying your bladder? Carry templates or other aids, to collect urine?	Are you losing bowel movements unintentionally? Do you have problems holding back intestinal wind or other problems with your intestines? Are there problems with dirty laundry?

**Table 4:** Representation of continence profiles ([25], p. 35).

Profile	Characteristic Urine- and Fecal Continence	Example Urinary Continence	Example Fecal Continence
**Continence**	No involuntary loss of urine and/or no involuntary loss of stool. No personal support necessary. No aids.	The excretion of urine and stool occurs randomly in an appropriate place at an appropriate time.	
**Independent reached Continence**	No involuntary loss of urine and/or no involuntary loss of stool. No personal support necessary. Independent implementation of measures.	People who are independent in the management and use of medication, mobile toilet aids, intermittent self-catheterization or in carrying out training measures (e.g., bladder training) and who do not have involuntary urine loss.	People who are involved in the management and use of medication, intestinal education, aids and/or in the implementation of training measures are independent and do not have involuntary loss of stool.
**Dependent reached Continence**	No involuntary loss of urine and/or no involuntary loss of stool. Personnel support necessary for the implementation of measures.	People for whom the management and use of medication or the management and use of other continence maintenance measures are carried out by another person or for whom assisted toileting is carried out at individual/set times.	
**Independent compensated Incontinence**	Involuntary loss of urine and/or no involuntary loss of stool No personal support necessary.	People who suffer from involuntary urinary incontinence and are able to manage and use compensatory measures such as absorbent aids, condom catheters, the care of a suprapubic or transurethral indwelling catheter and its drainage into appropriate leg or bed bags or catheter valves.	People who suffer from involuntary fecal incontinence and are able to manage and apply compensatory measures such as absorbent aids, other aids such as intra-anal incontinence products (anal tampons or plugs) or anal irrigation independently.
**Dependent compensated Incontinence**	Involuntary loss of urine and/or no involuntary loss of stool Personal support necessary.	Support and application of compensatory measures will be handled by another person.	
**Not compensated Incontinence**	Personal support and therapeutic or care measures are not being used.	Applies for to those affected who do not want to talk about their incontinence and do not accept personal support or aids, or who do not accept them due to cognitive impairment, or who remove the aids.	

**Table 5:** Example representation of yellow and red flags [33].

	Urine Excretion	Fecal Excretion
**Yellow flags**	Delirium Infections Medication Fecal incontinence	Delirium Infections Medication Increased diarrhea
**Red flags**	Hematuria Dysuria Paresis/paresthesia Overflow symptoms Feeling of pressure in the vagina	Pain during bowel movements Bleeding during bowel movements Blood deposits

## Data Availability

No new data were created or analyzed in this study. Data sharing is not applicable to this article.

## Supplementary Materials

The following supporting information can be downloaded at: https://www.mdpi.com/article/10.3390/healthcare13212771/s1.
