# Supplementary material for: Advancing Evidence-Based Nursing: The Updated German Expert Standard on Continence Promotion
Source: Healthcare (Basel). 2025 Oct 31;13(21):2771. doi: 10.3390/healthcare13212771 (PMC12609654; doi:10.3390/healthcare13212771)
Supplement: Supplementary file 1 [file healthcare-13-02771-s001.zip › healthcare-3879010-supplementary.pdf]

Advancing Evidence-Based Nursing: The Updated German Expert Standard on Continence Promotion

Urinary Incontinence

Experiences and Coping

- How do individuals experience urinary incontinence?
- What impact does urinary incontinence have on the lives of affected individuals?
- What impact does the urinary incontinence of affected individuals have on the lives of their relatives or caregivers?
- What kinds of burdens are individuals with urinary incontinence confronted with?
- How does urinary incontinence affect the quality of life of affected individuals?
- What coping strategies do individuals develop in relation to their urinary incontinence?
- What are the economic consequences of urinary incontinence for affected individuals?
- What are the economic consequences of urinary incontinence for the healthcare system?

Nursing Interventions

- Is watchful waiting an appropriate intervention to promote continence?
- Is biofeedback, in addition to pelvic floor training, an appropriate intervention to promote continence?
- Are vaginal cones, in addition to pelvic floor training, an appropriate intervention to promote continence?
- Is magnetic stimulation therapy, in addition to pelvic floor training, an appropriate intervention to promote continence?
- Is vibration training, in addition to pelvic floor training, an appropriate intervention to promote continence?
- Which preventive measures are suitable for preventing urinary incontinence?

Assessment

- Which screening instruments are suitable for identifying individuals at risk of and/or with existing urinary incontinence?

- Which assessment instruments are suitable for a differentiated evaluation of existing urinary incontinence?
- Which aspects should be considered in the nursing anamnesis for individuals at risk of and/or with existing urinary incontinence?

Fecal Incontinence

Experiences and Coping

- How do individuals experience fecal incontinence?
- What impact does fecal incontinence have on the lives of affected individuals?
- What impact does the fecal incontinence of affected individuals have on the lives of their relatives or caregivers?
- What kinds of burdens are individuals with fecal incontinence confronted with?
- How does fecal incontinence affect the quality of life of affected individuals?
- What coping strategies do individuals develop in relation to their fecal incontinence?
- What are the economic consequences of fecal incontinence for affected individuals?
- What are the economic consequences of fecal incontinence for the healthcare system?

Nursing Interventions

- Which nursing interventions to promote continence are suitable for individuals with fecal incontinence in German-speaking countries?
- Which preventive measures are suitable for preventing fecal incontinence?

Assessment

- Which screening instruments are suitable for identifying individuals at risk of and/or with existing fecal incontinence?
- Which assessment instruments are suitable for a differentiated evaluation of existing fecal incontinence?
- Which aspects should be considered in the nursing anamnesis for individuals at risk of and/or with existing fecal incontinence?
- Is a defecation diary a useful addition to the nursing anamnesis for individuals with fecal incontinence?

Advancing Evidence-Based Nursing: The Updated German Expert Standard on Continence Promotion

- Is a stool analysis a useful addition to the nursing anamnesis for individuals with fecal incontinence?
- Which questionnaires are suitable for assessing symptom burden and distress in individuals with fecal incontinence?

Urinary and Fecal Incontinence

Nursing Counseling

- What are the effects of nursing counseling programs for individuals with urinary and/or fecal incontinence?
- Are nursing counseling interventions effective for individuals with urinary and/or fecal incontinence?

Digital Assistive Technologies

- Which digital assistive technologies exist in the context of urinary and/or fecal incontinence?
